# A pivotal role of BEX1 in liver progenitor cell expansion in mice

**DOI:** 10.1186/s13287-018-0905-2

**Published:** 2018-06-15

**Authors:** Yuting Gu, Weiting Wei, Yiji Cheng, Bing Wan, Xinyuan Ding, Hui Wang, Yanyun Zhang, Min Jin

**Affiliations:** 1 Pediatric Institute of Soochow University, Institutes for Translational Medicine, Soochow University, Suzhou, Jiangsu China; 2 Department of Pediatric Surgery, Xinhua Hospital Affiliated to Shanghai Jiao Tong University School of Medicine, Shanghai, China; 3 Shanghai Institutes for Biological Sciences, Chinese Academy of Sciences, 320 Yueyang Road, Shanghai, 200031 China; 4 Department of Pharmacy, the Affiliated Suzhou Municipal Hospital, Nanjing Medical University, Suzhou, Jiangsu China

**Keywords:** Liver progenitor cells, Brain expressed X-linked 1, Expansion, Peroxisome proliferator-activated receptor gamma

## Abstract

**Background:**

The activation and expansion of bipotent liver progenitor cells (LPCs) are indispensable for liver regeneration after severe or chronic liver injury. However, the underlying molecular mechanisms regulating LPCs and LPC-mediated liver regeneration remain elusive.

**Methods:**

Hepatic brain-expressed X-linked 1 (BEX1) expression was evaluated using microarray screening, real-time polymerase chain reaction, immunoblotting and immunofluorescence. LPC activation and liver injury were studied following a choline-deficient, ethionine-supplemented (CDE) diet in wild-type (WT) and *Bex1*^−/−^ mice. Proliferation, apoptosis, colony formation and hepatic differentiation were examined in LPCs from WT and *Bex1*^−/−^ mice. Peroxisome proliferator-activated receptor gamma was detected in *Bex1*-deficient LPCs and mouse livers, and was silenced to analyse the expansion of LPCs from WT and *Bex1*^−/−^ mice.

**Results:**

Hepatic BEX1 expression was increased during CDE diet-induced liver injury and was highly elevated primarily in LPCs. *Bex1*^−/−^ mice fed a CDE diet displayed impaired LPC expansion and liver regeneration. *Bex1* deficiency inhibited LPC proliferation and enhanced LPC apoptosis in vitro. Additionally, *Bex1* deficiency inhibited the colony formation of LPCs but had no effect on their hepatic differentiation. Mechanistically, BEX1 inhibited peroxisome proliferator-activated receptor gamma to promote LPC expansion.

**Conclusion:**

Our findings indicate that BEX1 plays a pivotal role in LPC activation and expansion during liver regeneration, potentially providing novel targets for liver regeneration and chronic liver disease therapies.

**Electronic supplementary material:**

The online version of this article (10.1186/s13287-018-0905-2) contains supplementary material, which is available to authorized users.

## Background

The liver is the largest internal organ in the human body and is vital for the metabolism of nutrients, glycogen storage, drug detoxification, bile secretion, the synthesis of various plasma proteins and immune regulation [1, 2]. Due to its large volume, complicated functions and anatomical structure, the liver is rather susceptible to damage, pathogen infection and other disorders, such as hepatitis, fibrosis, cirrhosis and even tumours. In response to injury, the liver restores its parenchymal mass through mature liver parenchymal cells, hepatocytes and cholangiocytes. However, parenchymal cell-mediated regeneration is impaired following prolonged or severe liver injury, and ductular reactions (formation of ductular structures) developing in the liver and liver progenitor cells (LPCs) are assumed to be responsible for repairing liver damage [3–5].

LPCs, also known as ‘oval cells’, are a stem cell population within the liver that displays an ovoid nucleus and scant cytoplasm [2]. Upon massive liver injury and persistent loss of hepatocytes, LPCs can be activated to proliferate and migrate into the hepatic lobule where they differentiate into hepatocytes and biliary epithelial cells for liver regeneration [6]. A correlation has been shown between the extent of liver disease and the magnitude of the accompanying LPC response [7]. LPC expansion occurs in many human liver diseases and experimental animal models [8–10], and LPCs are considered potential targets for liver cell transplantation and therapeutic liver repopulation [11, 12]. Various cytokines, extracellular matrix components and growth factors, such as Wnt, Notch and fibroblast growth factor (FGF), have been demonstrated to be involved in LPC activation and expansion for liver regeneration through the activation of multiple cellular signalling pathways [8, 10, 13–16]. However, the mechanisms regulating LPC activation and expansion remain to be elucidated. Thus, it is of great importance to better understand the mechanisms governing LPC behaviour to potentially develop novel therapeutic strategies for pathological liver injury.

Brain-expressed X-linked 1 (BEX1), an adaptor or modulator of intracellular signalling, was first characterized with reduced expression in F9 teratocarcinoma cells following retinoic acid treatment [17]. BEX1 is involved in P75 neurotrophin receptor signalling, regulating the cell cycle and differentiation of neural stem cells [18, 19]. BEX1 expression was subsequently reported to be upregulated in spinal cord motor neurons and was required for neurons to recover from injury [20]. BEX1 is also associated with skeletal muscle regeneration after injury [21], suggesting potential effects of BEX1 on tissue regeneration. Additionally, a role for *Bex1* as a marker for hepatocyte differentiation/dedifferentiation processes and tumour formation was identified [22]. However, no studies have examined the role of BEX1 in LPC-mediated liver regeneration.

Here, we identified that hepatic BEX1 expression was increased during choline-deficient, ethionine-supplemented (CDE) diet-induced liver injury and was elevated to a high level primarily in LPCs. Interestingly, *Bex1* deficiency severely impaired LPC expansion and liver regeneration in CDE-induced liver injury by inhibiting the proliferation of and enhancing the apoptosis of LPCs, indicating a critical role for BEX1 in LPC activation. Additionally, BEX1 was required for the colony formation of LPCs but not for their hepatic differentiation. Furthermore, BEX1 inhibited peroxisome proliferator-activated receptor gamma (PPARG), which contributed to the promoting effects of BEX1 on LPC expansion. Our findings identify BEX1 as a critical regulator of LPC activation and expansion during liver injury and indicate that BEX1 may serve as a novel target for liver regeneration and chronic liver disease therapies.

## Methods

### Mice and animal models

C57BL/6 mice were purchased from the Shanghai Laboratory Animal Center of the Chinese Academy of Sciences. 129Sv/Ev wild-type (WT) mice were obtained from the Shanghai Xipuer-Bikai Laboratory Animal Limited Company. *Bex1*-deficient (*Bex1*^*−*/−^) mice were kindly provided by Professor Frank L. Margolis, University of Maryland, Baltimore, MD, USA [21]. Female mice 6–8 weeks old were administered a CDE diet (TROPHIC, Nantong, China) supplemented with 0.15% (w/v) d,l-ethionine (Sigma-Aldrich, St. Louis, MO, USA) in the drinking water for 3 weeks [23], while control mice received normal chow and drinking water. Rosiglitazone (TCI, Tokyo, Japan) or GW9662 (dose of 2 mg/kg; Selleckchem, Houston, TX, USA) was administered to mice via intraperitoneal injection every second day, for a dose of 50 mg/kg, and dimethyl sulphoxide (DMSO; Sigma-Aldrich) was injected into control mice. All mice were maintained under specific pathogen-free conditions in the vivarium of Shanghai Jiao Tong University School of Medicine. All animal procedures were approved by the Animal Welfare & Ethics Committee of Shanghai Jiao Tong University School of Medicine.

### Microarray

Total RNA was extracted from samples using TRIzol reagent (Invitrogen, Carlsbad, CA, USA). RNA integrity was checked using the Agilent Bioanalyzer 2100 system (Agilent Technologies, Santa Clara, CA, USA). Total RNA was purified using the RNeasy kit and RNase-Free DNase Set (QIAGEN, Hilden, Germany). RNA labelling and microarray hybridization were performed according to the Affymetrix expression analysis technical manual (Biotechnology Corporation, Shanghai, China). The arrays were scanned using the GeneChip scanner 3000 system (Affymetrix, Santa Clara, CA, USA) and Command Console software 3.1 (Affymetrix) using default settings. Raw data were normalized using the MAS 5.0 algorithm of Gene Spring software 11.0 (Agilent Technologies).

### Quantitative real-time polymerase chain reaction (PCR)

Total RNA was extracted from tissues or cells and was reverse transcribed using a reverse transcription system (TaKaRa, Shiga, Japan). Quantitative real-time polymerase chain reaction was performed using the SYBR Green PCR mix (Roche, Basel, Switzerland) in the ViiA™ 7 Real-Time PCR System (Applied Biosystems, Waltham, MA, USA). *Actb* was used as an internal control to normalize for differences in the amount of total RNA in each sample. The primer sequences are listed as follows: *Bex1*, forward 5′-ATGGAGTCCAAAGATCAAGGCG-3′ and reverse 5′-CTGGCTCCCTTCTGATGGTA-3′; *Epcam*, forward 5′-GATCATCGCTGTCATTGTGG-3′ and reverse 5′-CACGGCTAGGCATTAAGCTC-3′; *Afp*, forward 5′-CCCTCATCCTCCTGCTACATT-3′ and reverse 5′-CGGAACAAACTGGGTAAAGGT-3′; *Prom1*, forward 5′-GGAAAAGTTGCTCTGCGAAC-3′ and reverse 5′-TCTCAAGCTGAAAAGCAGCA-3′; *Myc*, forward 5′-ACTCGCCTCACTCAGCTCCC-3′ and reverse 5′-ACCGTCCGCTCACTCCCTCT-3′; *Cdkn1a*, forward 5′-CCTGGTTCCTTGCCACTTCTT-3′ and reverse 5′-CTGTTCTAGGCTGTGACTGCTTC-3′; *Bcl-2*, forward 5′-ATGTGTGTGGAGAGCGTCAACC-3′ and reverse 5′-TGAGCAGAGTCTTCAGAGACAGCC-3′; *Alb*, forward 5′-TGGGTAACCTTTCTCCTCCTCC-3′ and reverse 5′-CACTCTTGTGTGCTTCTCGGC-3′; *G6pc*, forward 5′-CATCAATCTCCTCTGGGTGGC-3′ and reverse 5′-CGTTGCTGTAGTAGTCGGTGTCC-3′; *Krt19*, forward 5′-ACCCTCCCGAGATTACAACCAC-3′ and reverse 5′-CAAGGCGTGTTCTGTCTCAAAC-3′; *Krt7*, forward 5′-AGGAGATCAACCGACGCAC-3′ and reverse 5′-GTCTCGTGAAGGGTCTTGAGG-3′; and *Pparg*, forward 5′-CCACAGTTGATTTCTCCAGCATTTC-3′ and reverse 5′-CAGGTTCTACTTTGATCGCACTTTG-3′. All primers were synthesized by Sangon Biotech (Shanghai, China).

### Immunoblotting

Tissues or cells were harvested and lysed with ice-cold RIPA buffer (Beyotime, Haimen, China) containing protease and phosphatase inhibitors (Roche). Lysates were clarified by centrifugation at 15,000 × *g* for 30 min. The protein concentration of the supernatant fraction was determined by the Bradford assay (Thermo Fisher Scientific, Waltham, MA, USA). Protein samples were diluted in 4× SDS loading buffer (TaKaRa), heated to 95 °C for 5 min and separated in a 10% SDS-polyacrylamide gel. The proteins were electroblotted onto polyvinylidene fluoride membranes and incubated for 1 h in 5% bovine serum albumin in phosphate-buffered saline (PBS) or non-fat dry milk dissolved in PBS containing 0.1% Tween-20 (PBST) at room temperature. The blotting membranes were incubated with primary antibodies to BEX1 (kindly provided by Professor Frank L. Margolis), CDKN1a, B-cell lymphoma-2 (Bcl-2), poly ADP-ribose polymerase (PARP), cleaved PARP and PPARG (Cell Signaling Technology, Danvers, MA, USA) overnight at 4 °C, extensively washed in PBST, incubated with HRP-conjugated secondary antibody (Cell Signaling Technology) for 1 h at room temperature and washed again with PBST. The blotting membranes were developed with chemiluminescent reagents (Millipore, Billerica, MA, USA) according to the manufacturer’s instructions. The densitometry of the bands was quantified using ImageJ software.

### Immunofluorescence (IF)

Liver tissues were dissected from mice and were fixed for 4 h in 4% paraformaldehyde (PFA), followed by incubation overnight in 30% sucrose before embedding in OCT. Frozen blocks were cut into 5-μm sections and stained as described previously [24, 25]. The primary antibodies included anti-BEX1 (kindly provided by Professor Frank L. Margolis), anti-epithelial cell adhesion molecule (EpCAM) and anti-Ki67 (Abcam, Cambridge, MA, USA). The secondary antibody was Alexa 488-conjugated and Alexa 555-conjugated antibodies (Invitrogen). The slides were mounted and examined by confocal microscopy (ZEISS, Oberkochen, Germany).

### Histological analysis

Liver tissues were dissected from mice and were immediately fixed in 4% PFA and embedded in paraffin. Paraffin-embedded 5-μm sections of the liver were stained with haematoxylin and eosin (H&E), and frozen 5-μm sections of the liver were stained with Oil Red O, and the sections were then examined by light microscopy.

### LPC isolation and culture

LPCs were isolated as described previously [24]. Briefly, liver tissues were removed from CDE diet-fed mice after in-situ perfusion using a two-step collagenase perfusion method and then were incubated with 1 mg/ml collagenase D (Roche) and 1 mg/ml pronase (Roche) at 37 °C for 30 min. Non-parenchymal cells (NPCs) were separated from hepatocytes by repeated low-speed centrifugation. Next, NPCs were collected and suspended in a discontinuous gradient of 20% and 50% Percoll (GE Healthcare, Pittsburgh, PA, USA) and centrifuged continuously at 1400 × *g* for 20 min to enrich LPCs. The enriched LPCs were labelled with APC-conjugated anti-EpCAM and FITC-conjugated anti-CD45 (eBioscience, San Diego, CA, USA) antibodies, and EpCAM^+^CD45^−^ cells were isolated by fluorescence-activated cell sorting. These LPCs were cultured in type I collagen-coated dishes (BD Biosciences, San Jose, CA, USA). The standard culture medium for LPCs was DMEM/F12 (Gibco, Grand Island, NY, USA) supplemented with 10% foetal bovine serum, 1 μg/ml insulin (Sigma-Aldrich), 1 × 10^− 7^ mol/L dexamethasone (Sigma-Aldrich), 1% penicillin/streptomycin (Invitrogen), 50 ng/ml hepatocyte growth factor (PeproTech, Rocky Hill, NJ, USA), 20 ng/ml epidermal growth factor (PeproTech), 20 ng/ml FGF (PeproTech) and 1× Insulin–Transferrin–Selenium–Ethanolamine (Invitrogen).

### Flow cytometry

Cells were washed with PBS, and the pellets were resuspended in PBS with 2.5% foetal bovine serum at a concentration of 5 × 10^5^ cells per 50 μl. Cells were stained with a combination of APC-conjugated-EpCAM, APC-conjugated-CD49f, PE-conjugated-CD44 and FITC-conjugated-CD45 antibody (eBioscience) for 30 min at 4 °C and then analysed by flow cytometry using a BD FACSCalibur Flow Cytometry System (BD Biosciences). For apoptosis analysis, the cells were stained with annexin V and propidium iodide (PI; eBioscience) for 10 min and analysed by flow cytometry. For the bromodeoxyuridine (BrdU; BD Biosciences) assay, the cells were incubated with BrdU at a final concentration of 10 μM in the cell culture medium for 4 h. The cells were harvested and washed with PBS. After fixation and permeabilization, the cells were treated with 300 μg/ml DNase (Roche). The incorporated BrdU was stained with anti-BrdU-FITC antibody (BD Biosciences) and then analysed by flow cytometry.

### Cell Counting Kit-8 (CCK8) assay

Cell proliferation and viability were monitored using CCK8 (Dojindo, Kumamoto, Japan) according to the manufacturer’s instructions. The cells were seeded onto 96-well plates, and cell proliferation and viability were assessed at the indicated time points by measurement of the absorbance at 450 nm.

### Lentiviral vector construction

Oligonucleotides with the following nucleotide sequences were used to clone shRNA-encoding sequences into a lentiviral vector PLKO.1 puro, a gift from Bob Weinberg (Addgene, Cambridge, MA, USA): mouse BEX1 (sh*Bex1*), 5’-CCGGTTATGTAGATCTCTCCCTGTTCTCGAGAACAGGGAGAGATCTACATAATTTTTG-3′; and scrambled control (shNC), 5’-CCGGCCTAAGGTTAAGTCGCCCTCGCTCGAGCGAGGGCGACTTAACCTTAGGTTTTTG-3′ (synthesized by Sangon Biotech). High-titre lentiviral stocks were produced, and liver epithelial progenitor cells (LEPCs), an LPC cell line [26], were infected with scrambled control lentivirus (shNC) or lentivirus expressing shRNA inhibiting BEX1 (sh*Bex1*). Cells resistant to puromycin (2 μg/ml) were selected and passaged for further study. Production of high-titre lentiviral stocks and lentiviral stocks transfection were handled according to the manufacturer’s protocol.

### Clonogenic colony-forming assay

The cells were diluted and seeded at 200 cells per well of a six-well plate. After incubation for 10 days, the cells were washed with PBS, fixed with 4% PFA and stained with crystal violet. The numbers of visible colonies were counted.

### Hepatic differentiation induction

Hepatic differentiation was induced as described previously [27]. Briefly, LPCs were grown to confluence, washed with PBS and cultured in medium supplemented with 20% Matrigel (BD Biosciences), 40 ng/ml oncostatin M (R&D Systems, Minneapolis, MN, USA), 25 ng/ml hepatocyte growth factor, 25 ng/ml epidermal growth factor, and 10^− 7^ M dexamethasone for 7 days. Next, the cells were harvested for real-time PCR or subjected to periodic acid–Schiff (PAS) staining. Cells grown on chamber slides were fixed with 4% PFA for 30 min and washed with PBS. The slides were then incubated in periodic acid solution for 5 min and rinsed with several changes of distilled water. Finally, the slides were incubated in Schiff’s reagent (Sigma-Aldrich) for 15 min, washed in running tap water for 5 min, dehydrated and mounted with xylene-based mounting medium.

### Statistical analyses

Statistical analyses were performed using SPSS. All values are expressed as the mean ± SEM. Statistical significance was evaluated using an unpaired non-parametric test. *P* < 0.05 was considered significant.

## Results

### Hepatic BEX1 expression is upregulated in response to a CDE diet

**Fig. 1:**
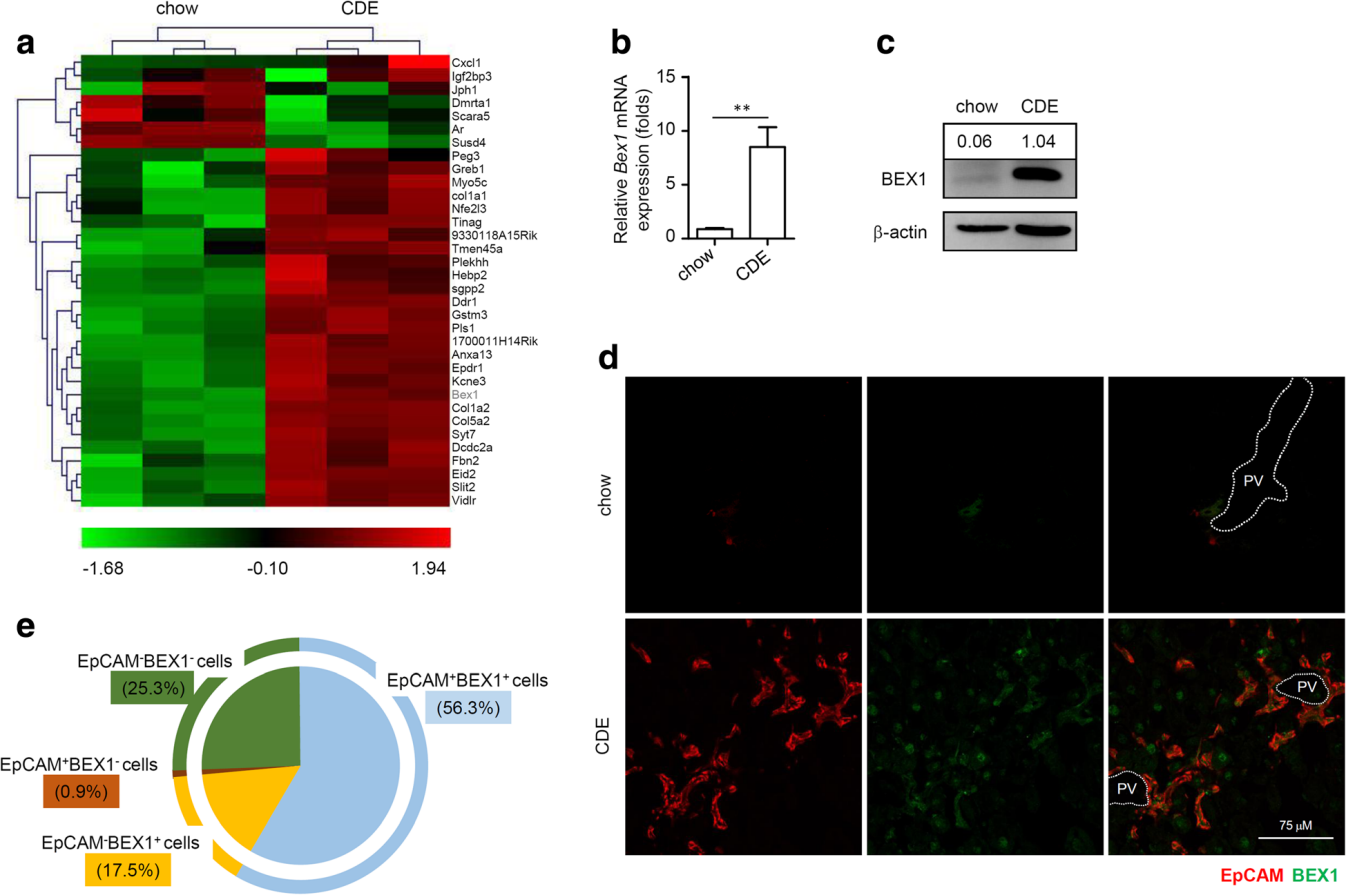
Hepatic BEX1 expression is upregulated in response to CDE diet. **a** Heat map representation of significantly changed genes in liver tissues of 3-week chow and CDE diet-fed mice (*n* = 3). **b** Expression of *Bex1* in liver tissues of 3-week chow and CDE diet-fed mice assessed by real-time PCR (mean ± SEM, *n* = 5, **P < 0.01, Mann–Whitney *U* test). **c** Immunoblotting analysis of BEX1 expression in liver lysates of 3-week chow and CDE diet-fed mice. **d** Mice fed chow or CDE diet for 3 weeks, livers excised and frozen sections prepared. Histologic specimens dual-stained for EpCAM (red) and BEX1 (green). IF staining examined by confocal microscopy. Scale bars, 75 μm. **e** Percentages of EpCAM^+^BEX1^+^ cells, EpCAM^+^BEX1^−^ cells, EpCAM^−^BEX1^+^ cells and EpCAM^−^BEX1^−^ cells in liver tissues of CDE diet-fed mice (*n* = 4). BEX1 brain-expressed X-linked 1, CDE choline-deficient, ethionine-supplemented, EpCAM epithelial cell adhesion molecule, PV portal vein

To investigate the molecular mechanisms regulating LPC activation and LPC-mediated liver regeneration, we fed mice a modified CDE diet to induce liver damage [23] and performed microarray analysis on the liver tissues of mice fed with chow and CDE diets. Based on the changes in the gene expression profiles, we found that *Bex1*, a previously unknown gene involved in liver regeneration, was greatly upregulated in the liver tissues of CDE diet-fed mice compared with those of chow diet-fed mice (Fig. 1a). We then assessed the mRNA level of *Bex1* in the liver tissues of mice fed with the chow and CDE diets by real-time PCR and found that hepatic *Bex1* mRNA was significantly higher in the CDE diet-fed mice (Fig. 1b). Immunoblotting analysis revealed a similar result for the protein level of hepatic BEX1 (Fig. 1c). We next examined whether BEX1 was expressed in LPCs. LPCs were induced around the portal vein by the CDE diet, which were identified by the expression of a specific marker, EpCAM [28]. We found that BEX1 was rarely expressed in the liver tissues of chow-fed mice, but was strongly expressed in EpCAM^+^ LPCs and some surrounding hepatocytes in CDE diet-fed mice (Fig. 1d, e). Altogether, our data showed that hepatic BEX1 expression was increased during CDE diet-induced liver injury and was elevated to a high level primarily in LPCs, implicating the essential role of BEX1 for LPC activation and LPC-mediated liver regeneration.

### BEX1 is critical for LPC expansion and liver regeneration in response to a CDE diet

**Fig. 2:**
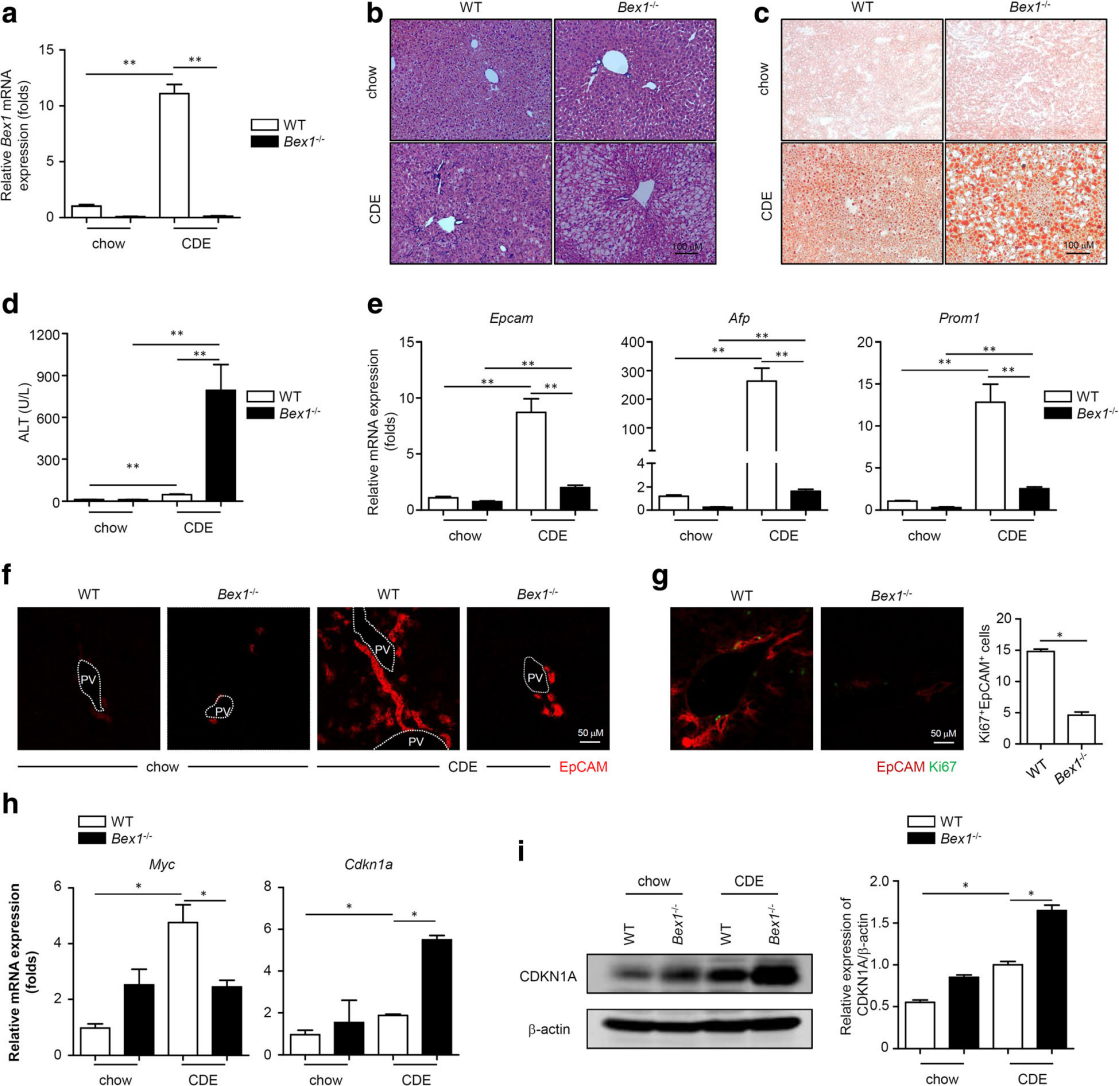
*Bex1* deficiency reduces LPC expansion and liver regeneration in response to CDE diet. **a** Expression of *Bex1* in liver tissues of 3-week chow and CDE diet-fed WT and *Bex1*^−/−^ mice measured by real-time PCR (mean ± SEM, *n* = 5, **P < 0.01, Mann–Whitney *U* test). **b, c** Histologic specimens of liver tissues from 3-week chow and CDE diet-fed WT and *Bex1*^−/−^ mice stained with H&E and Oil Red O. Scale bars, 100 μm. **d** Serum ALT levels of 3-week chow and CDE diet-fed WT and *Bex1*^−/−^ mice (mean ± SEM, *n* = 5, **P < 0.01, Mann–Whitney *U* test). **e** Expression of *Epcam*, *Afp* and *Prom1* in liver tissues of 3-week chow and CDE diet-fed WT and *Bex1*^−/−^ mice measured by real-time PCR (mean ± SEM, *n* = 5, **P < 0.01, Mann–Whitney *U* test). **f** IF staining of EpCAM (red) in liver tissues of 3-week chow or CDE diet-fed WT and *Bex1*^−/−^ mice. Results representative of three independent experiments with similar results. Scale bars, 50 μm. **g** IF staining of EpCAM (red) and Ki67 (green) in liver tissues of 3-week chow and CDE diet-fed WT and *Bex1*^−/−^mice. Scale bars, 50 μm. Quantification of Ki67^+^ EPCAM^+^ cells (mean ± SEM, *n* = 5, *P < 0.05, Mann–Whitney *U* test). **h** Expression of *Myc* and *Cdkn1a* in liver tissues of 3-week chow and CDE diet-fed WT and *Bex1*^−/−^ mice measured by real-time PCR (mean ± SEM, *n* = 4, *P < 0.05, Mann–Whitney *U* test). **i** Immunoblotting analysis of CDKN1A expression in liver tissues of 3-week chow or CDE diet-fed WT and *Bex1*^−/−^ mice. Quantifying densitometry of bands using ImageJ software (mean ± SEM, *n* = 4, *P < 0.05, Mann–Whitney *U* test). AFP α-fetoprotein, ALT alanine aminotransferase, BEX1 brain-expressed X-linked 1, CDE choline-deficient, ethionine-supplemented, EpCAM epithelial cell adhesion molecule, PROM1 prominin-1, PV portal vein, WT wild-type

To determine whether BEX1 functionally regulates LPC activation and LPC-mediated liver regeneration, we fed the chow or CDE diet to WT and *Bex1*^−/−^ mice [21]. *Bex1* deficiency in *Bex1*^−/−^ mice was confirmed by PCR with genomic DNA (Additional file 1: Figure S1), and *Bex1* mRNA expression was dramatically downregulated in the liver tissues of *Bex1*^−/−^ mice compared with those of WT mice fed with either the chow or CDE diet (Fig. 2a). Histological analysis showed that the livers of both chow diet-fed WT and *Bex1*^−/−^ mice were structurally integrated. Following the CDE diet, ductular reactions were induced in WT mice as reported previously but were not as obvious in *Bex1*^−/−^ mice (Fig. 2b). Furthermore, compared with CDE diet-fed WT mice, the livers of CDE diet-fed *Bex1*^−/−^ mice exhibited more serious macrovesicular steatosis, indicated by more macrovesicular fat droplets and fat accumulation (Fig. 2b, c). These CDE diet-fed *Bex1*^−/−^ mice also showed a dramatic increase in serum alanine aminotransferase (ALT) levels compared with the CDE diet-fed WT mice (Fig. 2d), indicating substantially more severe liver injury. These results suggest that *Bex1*^−/−^ mice have impaired liver regeneration capacity in response to the CDE diet.

To test LPC activation in BEX1-regulated liver regeneration, we first measured the expression levels of the LPC markers EpCAM, α-fetoprotein (AFP) and prominin-1 (PROM1) in the liver tissues of WT and *Bex1*^−/−^ mice fed with the chow or CDE diet. The mRNA levels of *Epcam*, *Afp* and *Prom1* were increased in the liver tissues of WT mice after the CDE diet (Fig. 2e). Notably, the liver tissues of the CDE diet-fed *Bex1*^−/−^ mice showed markedly lower mRNA levels of *Epcam*, *Afp* and *Prom1* than those of CDE diet-fed WT mice (Fig. 2e). IF staining demonstrated that after the CDE diet, *Bex1*^−/−^ mice had significantly lower numbers of EpCAM^+^ and Ki67^+^EpCAM^+^ LPCs than WT mice (Fig. 2f, g). Next, we tested the mRNA levels of the cell cycle regulators *Myc* and *Cdkn1a*, which regulate cellular proliferation, in liver tissues. As expected, the *Myc* transcript level was upregulated in CDE diet-fed WT mice but not in CDE diet-fed *Bex1*^−/−^ mice, while the cell cycle-dependent kinase inhibitor *Cdkn1a* level was upregulated in CDE diet-fed *Bex1*^−/−^ mice (Fig. 2h). CDKN1A induction was also assessed by immunoblotting analysis, which showed similar results (Fig. 2i). Taken together, these results indicate that BEX1 is required for LPC expansion and proliferation in the CDE diet-induced liver injury model.

### BEX1 promotes LPC proliferation and prevents LPC apoptosis in vitro

**Fig. 3:**
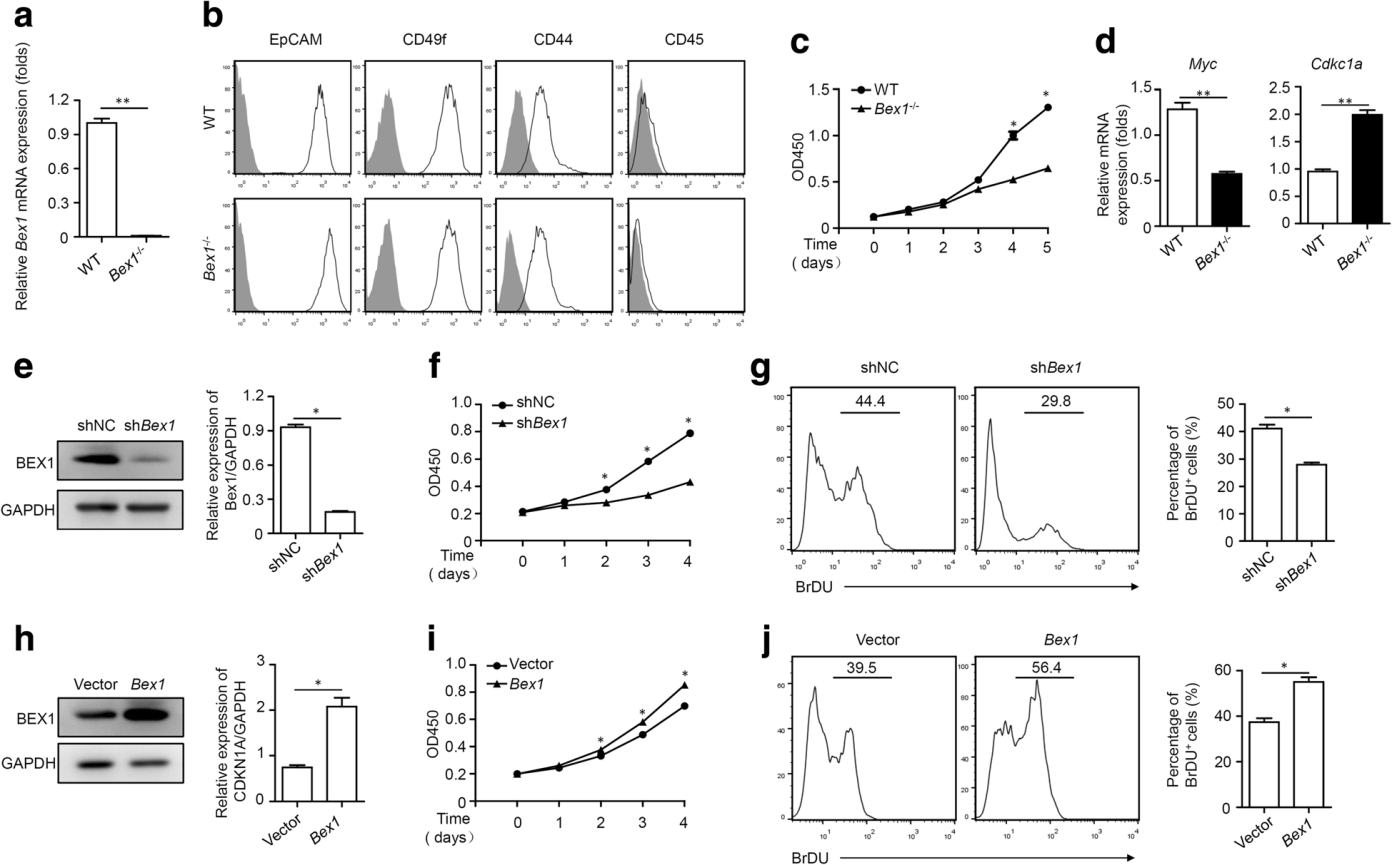
BEX1 promotes LPC proliferation in vitro. Primary LPCs isolated from 3-week CDE diet-fed WT and *Bex1*^−/−^ mice. **a** Real-time PCR analysis of *Bex1* expression in LPCs from WT and *Bex1*^−/−^ mice (mean ± SEM, *n* = 5, **P < 0.01, Mann–Whitney *U* test). **b** Flow cytometry analysis of EpCAM, CD49f, CD44 and CD45 in LPCs from WT and *Bex1*^−/−^ mice. Results representative of three independent experiments with similar results. **c** CCK8 assay showed proliferation of LPCs from WT and *Bex1*^−/−^ mice (mean ± SEM, *n* = 4, *P < 0.05, Mann–Whitney *U* test). **d** Real-time PCR analysis of *Myc* and *Cdkn1a* expression in LPCs from WT and *Bex1*^−/−^ mice (mean ± SEM, *n* = 5, **P < 0.01, Mann–Whitney *U* test). **e** Immunoblotting analysis of BEX1 expression in LEPCs infected with control lentivirus (shNC) or lentivirus expressing shRNA targeting BEX1 (sh*Bex1*).Quantifying densitometry of bands using ImageJ software (mean ± SEM, *n* = 4, *P < 0.05, Mann–Whitney *U* test). **f** CCK8 assay showed proliferation of shNC and sh*Bex1* LEPCs (mean ± SEM, *n* = 4, *P < 0.05, Mann–Whitney *U* test). **g** BrdU assay showed proliferation of shNC andsh*Bex1* LEPCs, and percentage of BrdU^+^ LEPCs analysed by flow cytometry (mean ± SEM, *n* = 4, *P < 0.05, Mann–Whitney *U* test). **h** *Bex1* overexpressed in LEPCs. BEX1 expression detected by immunoblotting analysis. Quantifying densitometry of bands using ImageJ software (mean ± SEM, *n* = 4, *P < 0.05, Mann–Whitney *U* test). **i** CCK8 assay showed proliferation of Vector and *Bex1* LEPCs (mean ± SEM, *n* = 4, *P < 0.05, Mann–Whitney *U* test). **j** BrdU assay showed proliferation of Vector and *Bex1* LEPCs, and percentage of BrdU^+^ LEPCs analysed by flow cytometry (mean ± SEM, *n* = 4, *P < 0.05, Mann–Whitney *U* test). BEX1 brain-expressed X-linked 1, BrdU bromodeoxyuridine, EpCAM epithelial cell adhesion molecule, GAPDH glyceraldehyde 3-phosphate dehydrogenase, WT wild-type

To further clarify the effect of BEX1 on LPC proliferation, we isolated EpCAM^+^CD45^−^ LPCs from WT and *Bex1*^−/−^ mice fed with the CDE diet. *Bex1* mRNA expression was significantly lower in LPCs from *Bex1*^−/−^ mice than in those from WT mice (Fig. 3a). LPCs from WT and *Bex1*^−/−^ mice displayed a similar phenotype, EpCAM^+^CD49f^+^CD44^+^CD45^−^ (Fig. 3b). However, in vitro proliferation assays showed that the proliferation of LPCs from WT mice was higher than those from *Bex1*^−/−^ mice (Fig. 3c). The decreased *Myc* expression and increased *Cdkn1a* mRNA expression were consistent with a lower proliferation of LPCs from *Bex1*^−/−^ mice (Fig. 3d). Meanwhile, *Bex1* was knocked down in LEPCs using a lentivirus expressing shRNA specific to BEX1 (sh*Bex1*). LEPCs infected with a lentivirus expressing scrambled shRNA (shNC) were used as controls (Fig. 3e). The proliferation ability of sh*Bex1*-LEPCs was also impaired (Fig. 3f, g). By contrast, the proliferation of BEX1-overexpressed LEPCs was enhanced, as shown by CCK8 and BrdU analysis (Fig. 3h–j).

**Fig. 4:**
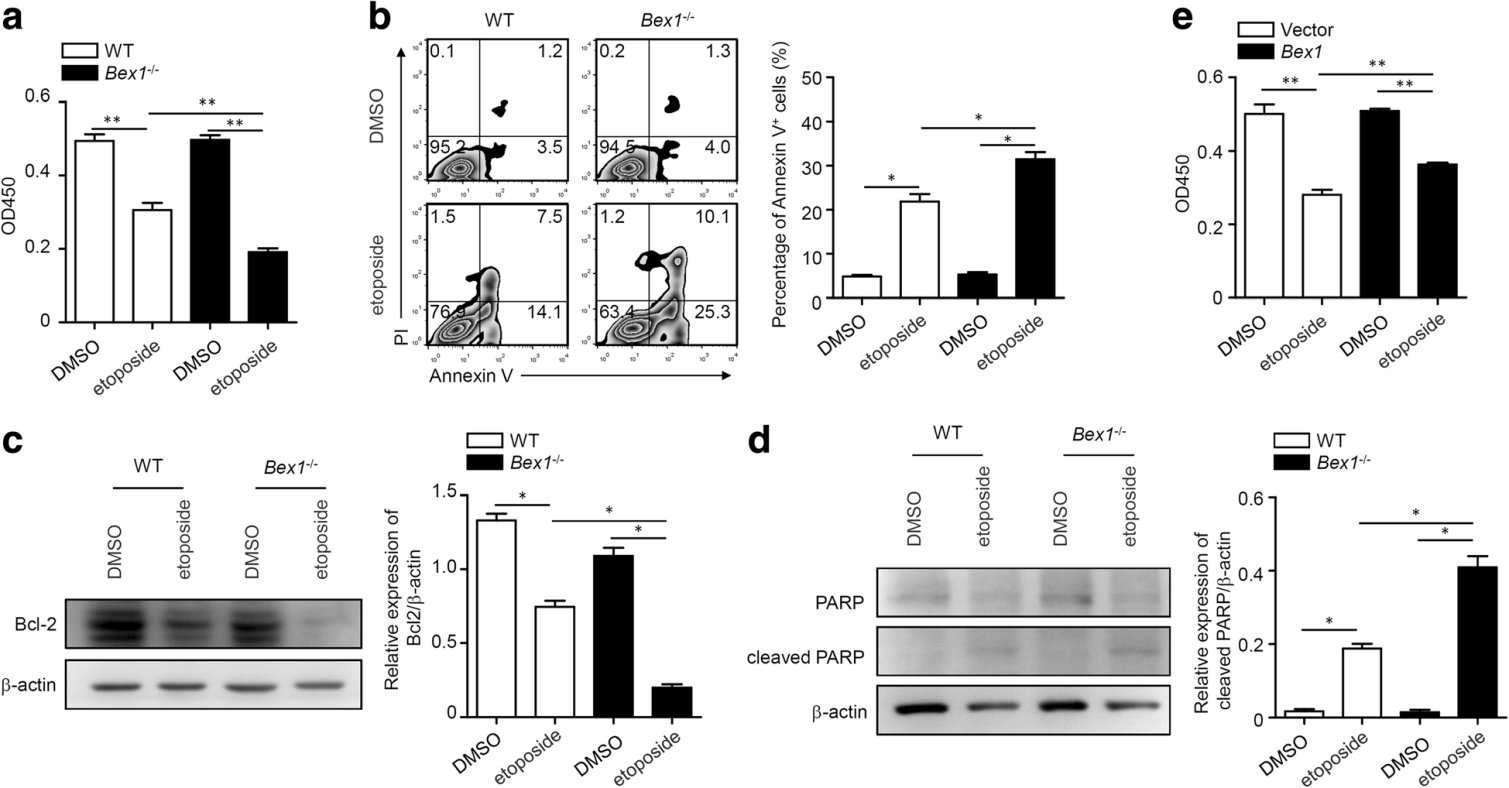
BEX1 prevents LPC apoptosis in vitro. Primary LPCs isolated from 3-week CDE diet-fed WT and *Bex1*^−/−^ mice as described in Methods. **a** LPCs from WT and *Bex1*^−/−^ mice treated with DMSO or 40 μM etoposide for 24 h, and cell viability quantified by CCK8 assay (mean ± SEM, *n* = 5, **P < 0.01, Mann–Whitney *U* test). **b** Cell apoptosis of LPCs from WT and *Bex1*^*−/−*^mice treated with DMSO or 40 μM etoposide for 24 h detected by annexin V/PI staining (mean ± SEM, *n* = 4, *P < 0.05, Mann–Whitney *U* test). **c, d** LPCs from WT and *Bex1*^−/−^ mice treated with DMSO or 40 μM etoposide for 24 h, and expression of Bcl-2, PARP and cleaved PARP detected by immunoblotting analysis. Quantifying densitometry of bands using ImageJ software (mean ± SEM, *n* = 4, *P < 0.05, Mann–Whitney *U* test). **e** Vector and *Bex1* LEPCs treated with DMSO or 40 μM etoposide for 24 h, and cell viability quantified by CCK8 assay (mean ± SEM, *n* = 5, **P < 0.01, Mann–Whitney *U* test). Bcl-2 B-cell lymphoma-2, BEX1 brain-expressed X-linked 1, DMSO dimethyl sulphoxide, PARP poly ADP-ribose polymerase, PI propidium iodide, WT wild-type

Reduced apoptosis alone, or combined with promoted proliferation, contributed to cellular expansion. We next tested whether the inhibition of BEX1 would affect LPC viability. LPCs from WT and *Bex1*^−/−^ mice were treated with etoposide, a cell apoptosis inducer [29], and cell viability was detected by CCK8 and annexin V/PI staining. As expected, both cell types displayed obvious cell apoptosis with etoposide treatment; however, the level of apoptosis in LPCs from *Bex1*^−/−^ mice was higher than that in LPCs from WT mice (Fig. 4a, b). The anti-apoptotic protein Bcl-2 was inhibited and the cleaved PARP was induced after etoposide treatment in LPCs from WT mice, and these effects were enhanced in LPCs from *Bex1*^−/−^ mice, also indicating enhanced apoptosis (Fig. 4c, d). In contrast, etoposide-induced cell apoptosis was alleviated in BEX1-overexpressed LEPCs (Fig. 4e). Taken together, our findings illustrate that BEX1 promotes proliferation and suppresses apoptosis of LPCs to augment cellular expansion and liver regeneration.

### BEX1 is not required for hepatic differentiation of LPCs

**Fig. 5:**
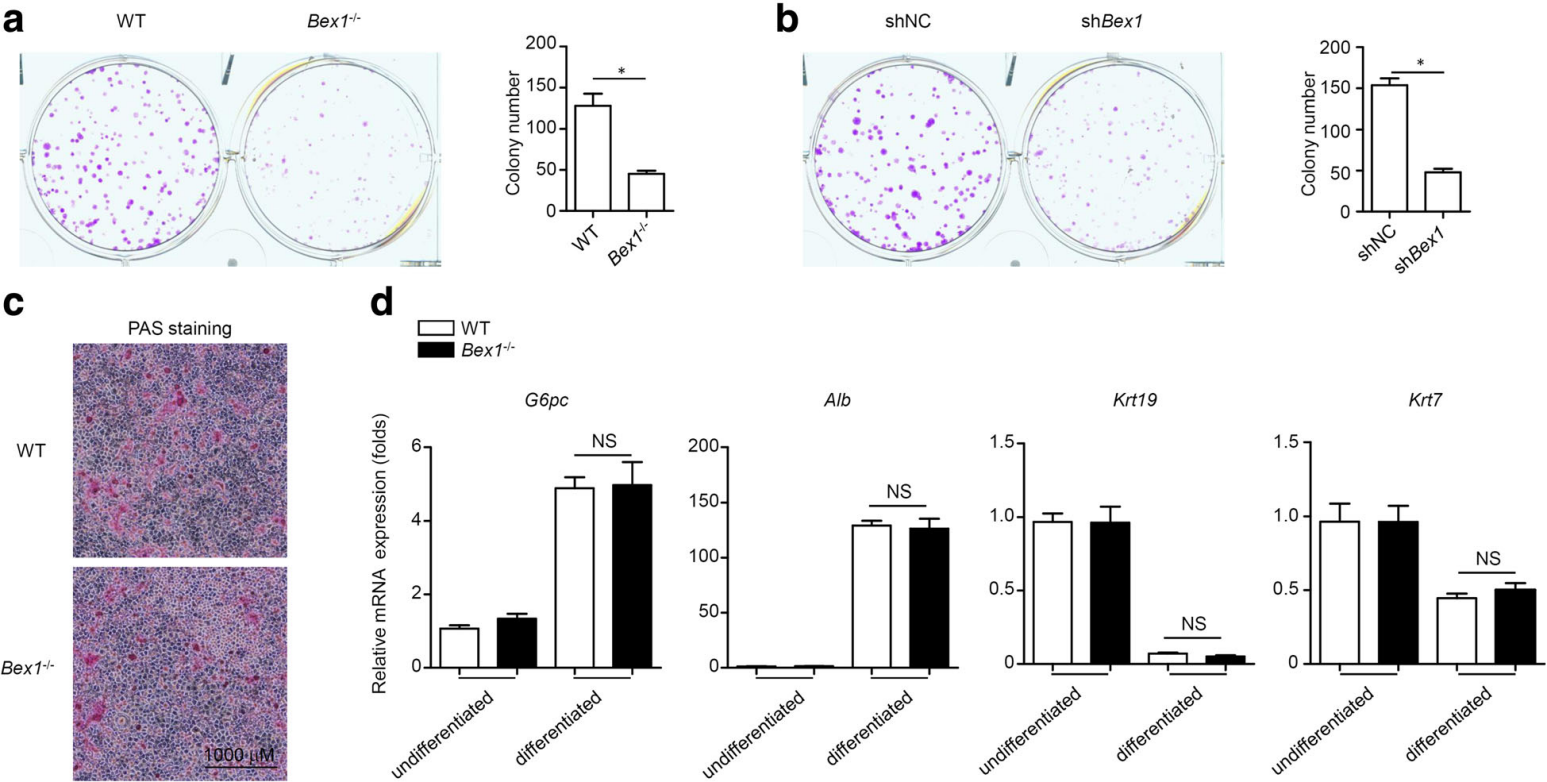
BEX1 has no impact on hepatic differentiation of LPCs. **a** Clonogenic colony-forming assay of cultured LPCs from WT and *Bex1*^−/−^ mice established. Number of colonies quantified (mean ± SEM, *n* = 4, *P < 0.05, Mann–Whitney *U* test). **b** Clonogenic colony-forming assay of shNC and sh*Bex1* LEPCs established. Number of colonies quantified (mean ± SEM, *n* = 4, *P < 0.05, Mann–Whitney *U* test). **c** LPCs from WT and *Bex1*^−/−^ mice cultured in hepatic differentiation medium for 7 days and subjected to PAS staining. Scale bars, 1000 μm. Results representative of three independent experiments with similar results. **d** Real-time PCR analysis of *G6pc*, *Alb, Krt19* and *Krt7* expression (mean ± SEM, *n* = 5, ^NS^*P* > 0.05, Mann–Whitney *U* test). ALB albumin, BEX1 brain-expressed X-linked 1, G-6-Pase glucose 6-phosphatase, NS not significant, PAS periodic acid–Schiff, sh*Bex1* lentivirus expressing shRNA specific to BEX1, shNC lentivirus expressing scrambled shRNA, WT wild-type

Self-renewal and differentiation are two characteristics of stem/progenitor cells, including LPCs, and these abilities of LPCs play critical roles in their activation and expansion during liver regeneration [3]. The efficiency of LPC self-renewal was assessed by the rate of colony formation in the clonogenic colony-forming assay. The colony formation of LPCs from *Bex1*^−/−^ mice was reduced compared with those from WT mice (Fig. 5a). Similar results were also detected in shNC and sh*Bex1* LEPCs (Fig. 5b). Furthermore, we performed PAS staining for glycogen deposition to detect the hepatic differentiation of LPCs. LPCs from both WT and *Bex1*^−/−^ mice could differentiate into hepatocytes upon induction with rare differences (Fig. 5c). We also analysed the hepatocyte markers glucose 6-phosphatase (G-6-Pase) and albumin (ALB) and the cholangiocyte markers cytokeratin-19 (CK-19) and CK-7. Real-time PCR analysis demonstrated that hepatic differentiation-induced upregulation of *G6pc* and *Alb* mRNA expression, as well as downregulation of *Krt19* and *Krt7* mRNA expression, showed no changes (Fig. 5d). These results indicate that BEX1 is required for the colony formation of LPCs but not for their hepatic differentiation.

### BEX1 inhibits PPARG to regulate LPC expansion

**Fig. 6:**
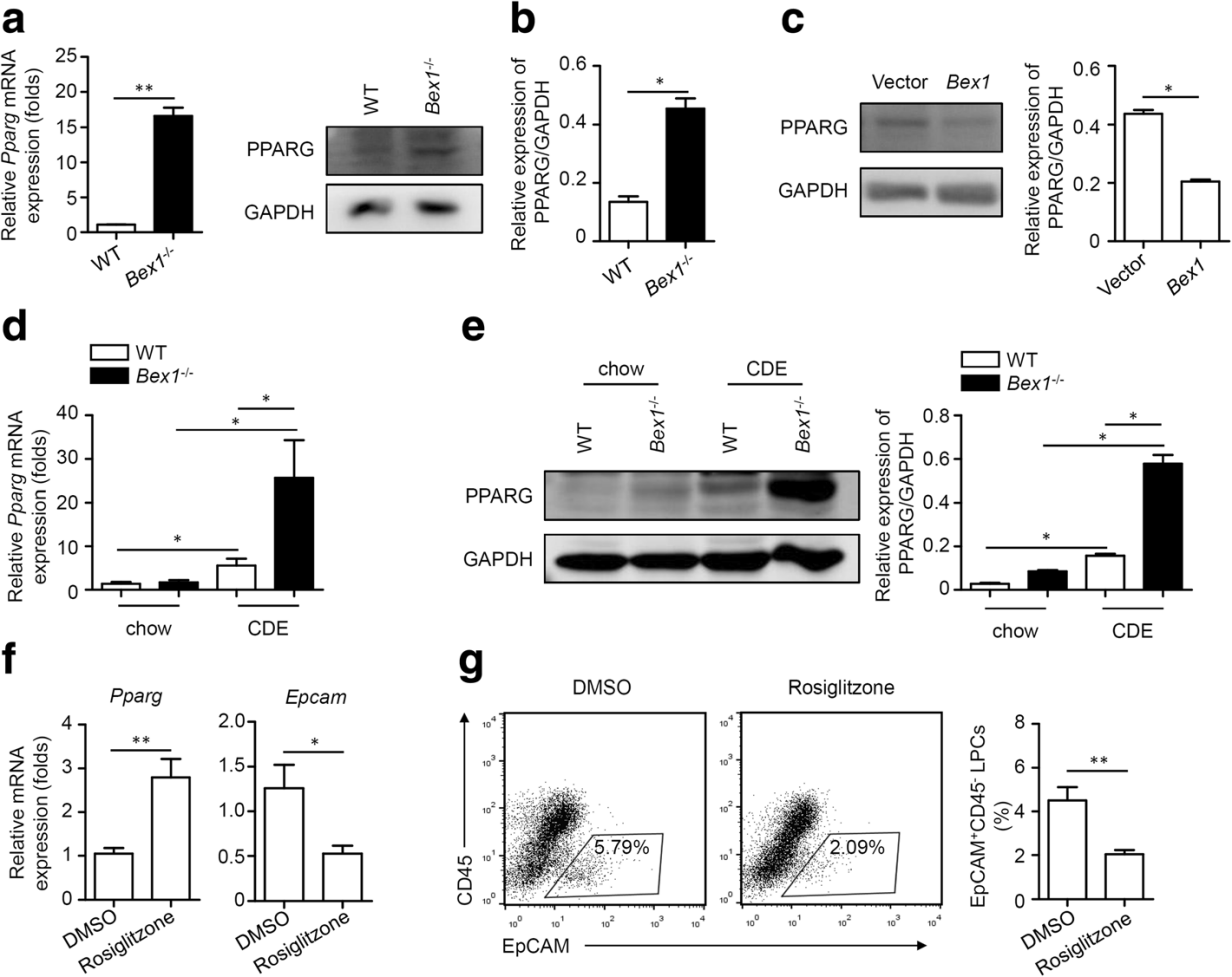
BEX1 inhibits PPARG in LPCs and liver tissues in response to CDE diet. **a** Real-time PCR analysis of *Pparg* mRNA level in LPCs from WT and *Bex1*^−/−^ mice (mean ± SEM, *n* = 6, **P < 0.01, Mann–Whitney *U* test). **b** Immunoblotting analysis of PPARG protein level in LPCs from WT and *Bex1*^−/−^ mice. Quantifying densitometry of bands using ImageJ software (mean ± SEM, *n* = 4, *P < 0.05, Mann–Whitney *U* test). **c** Immunoblotting analysis of PPARG protein level in Vector and *Bex1* LEPCs. Quantifying densitometry of bands using ImageJ software (mean ± SEM, *n* = 4, *P < 0.05, Mann–Whitney *U* test). **d** Real-time PCR analysis of *Pparg* expression in liver tissues of 3-week chow and CDE diet-fed WT and *Bex1*^−/−^ mice (mean ± SEM, *n* = 4–5, *P < 0.05, Mann–Whitney *U* test). **e** Immunoblotting analysis of PPARG protein level in liver tissues of 3-week chow and CDE diet-fed WT and *Bex1*^−/−^ mice. Quantifying densitometry of bands using ImageJ software (mean ± SEM, *n* = 4, *P < 0.05, Mann–Whitney *U* test). **f** Real-time PCR analysis of *Pparg* and *Epcam* expression in liver tissues of 3-week CDE diet-fed WT mice with DMSO or rosiglitazone treatment (mean ± SEM, *n* = 5, *P < 0.05, **P < 0.01, Mann–Whitney *U* test). **g** Flow cytometry analysis of percentage of EPCAM^+^CD45^−^ LPCs in NPCs in livers of 3-week-old CDE diet-fed WT mice with DMSO or rosiglitazone treatment (mean ± SEM, *n* = 5, **P < 0.01, Mann–Whitney *U* test). BEX1 brain-expressed X-linked 1, CDE choline-deficient, ethionine-supplemented, DMSO dimethyl sulphoxide, EpCAM epithelial cell adhesion molecule, GAPDH glyceraldehyde 3-phosphate dehydrogenase, LPC liver progenitor cell, PPARG peroxisome proliferator-activated receptor gamma, WT wild-type

PPARG, a nuclear receptor, plays important roles in LPC cell growth and ability during liver injury [30–32]. Thus, the roles of PPARG in BEX1-regulated LPC expansion were explored. PPAPG expression in LPCs from *Bex1*^−/−^ mice was higher than that in LPCs from WT mice, as detected by real-time PCR and immunoblotting analysis (Fig. 6a, b). By contrast, the protein level of PPAPG was decreased in BEX1-overexpressed LEPCs (Fig. 6c). The mRNA and protein levels of PPARG were also significantly increased in the liver tissues of CDE diet-fed *Bex1*^−/−^ mice compared with those in the liver tissues of CDE diet-fed WT mice (Fig. 6d, e). We next administered rosiglitazone, a PPARG-specific agonist, to CDE diet-fed WT mice to confirm the role of PPARG as a regulator for LPC expansion in liver regeneration. As expected, rosiglitazone increased *Pparg* expression and decreased *Epcam* expression in CDE diet-fed WT mice (Fig. 6f). Flow cytometry analysis demonstrated that rosiglitazone treatment reduced the percentage of EpCAM^+^ LPCs in the liver tissues of CDE diet-fed WT mice (Fig. 6g).

**Fig. 7:**
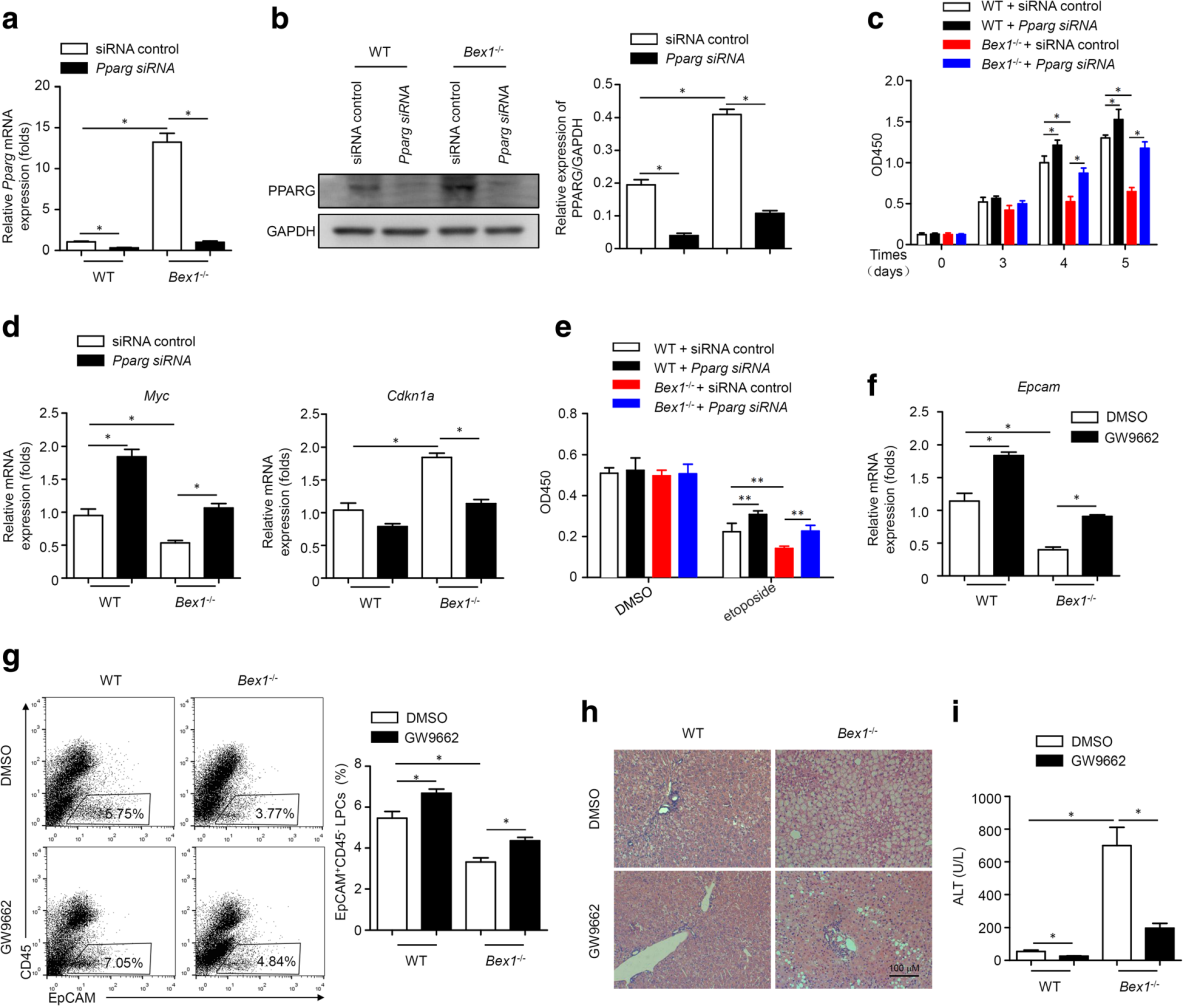
BEX1 inhibits PPARG to promote LPC expansion. LPCs from WT and *Bex1*^−/−^ mice transfected with siRNA control or siRNA against *Pparg*. **a** Expression of *Pparg* assessed by real-time PCR (mean ± SEM, *n* = 4, *P < 0.05, Mann–Whitney *U* test). **b** Immunoblotting analysis of PPARG expression. Quantifying densitometry of bands using ImageJ software (mean ± SEM, *n* = 4, *P < 0.05, Mann–Whitney *U* test).**c** CCK8 assay showed proliferation of LPCs from WT and *Bex1*^−/−^ mice transfected with siRNA control or siRNA against *Pparg* (mean ± SEM, *n* = 4, *P < 0.05, Mann–Whitney *U* test). **d** Real-time PCR analysis of *Myc* and *Cdkn1a* expression in LPCs from WT and *Bex1*^−/−^ mice transfected with siRNA control or siRNA against *Pparg* (mean ± SEM, *n* = 4, *P < 0.05, Mann–Whitney *U* test). **e** LPCs from WT and *Bex1*^−/−^ mice transfected with siRNA control or siRNA against *Pparg* cultured in presence of DMSO or40 μM etoposide for 24 h. Cell viability quantified by CCK8 assay (mean ± SEM, *n* = 5, **P < 0.01, Mann–Whitney *U* test). **f, g** Real-time PCR analysis of *Epcam* expression in liver tissues and flow cytometry analysis of percentage of EPCAM^+^CD45^−^ LPCs in NPCs in livers of 3-week CDE diet-fed WT and *Bex1*^−/−^ mice with DMSO or GW9662 treatment (mean ± SEM, *n* = 4, *P < 0.05, Mann–Whitney *U* test). **h** Histologic specimens of liver tissues from 3-week CDE diet-fed WT and *Bex1*^−/−^ mice with DMSO or GW9662 treatment stained with H&E. Scale bars, 100 μm. **i** Serum ALT levels of 3-week CDE diet-fed WT and *Bex1*^−/−^ mice with DMSO or GW9662 treatment (mean ± SEM, *n* = 4, *P < 0.05, Mann–Whitney *U* test). ALT alanine aminotransferase, BEX1 brain-expressed X-linked 1, DMSO dimethyl sulphoxide, EpCAM epithelial cell adhesion molecule, GAPDH glyceraldehyde 3-phosphate dehydrogenase, GW9662 PPARG antagonist, LPC liver progenitor cell, PPARG peroxisome proliferator-activated receptor gamma, WT wild-type

To further determine whether PPARG is involved in BEX1-mediated LPC expansion, we silenced *Pparg* in LPCs from CDE diet-fed WT and *Bex1*^−/−^ mice. siRNA specific to *Pparg* reduced the mRNA and protein levels of PPARG in LPCs (Fig. 7a, b). Silencing *Pparg* in LPCs from WT mice enhanced LPC proliferation (Fig. 7c). Moreover, silencing *Pparg* reversed the inhibited proliferation of LPCs from *Bex1*^−/−^ mice (Fig. 7c). The increased *Myc* expression and decreased *Cdkn1a* expression were consistent with the enhanced proliferation of LPCs from both WT and *Bex1*^−/−^ mice (Fig. 7d). The apoptosis of LPCs was also analysed. As shown in Fig. 7e, *Pparg* knockdown increased cell viability in LPCs with etoposide treatment from both WT and *Bex1*^−/−^ mice. More importantly, we next administered CDE diet-fed WT and *Bex1*^*−/−*^mice with the PPARG antagonist GW9662, to confirm the role of PPARG in BEX1-regulated LPC expansion in liver regeneration. GW9662 increased *Epcam* expression and the percentage of EpCAM^+^ LPCs in the liver tissues of CDE diet-fed WT mice, and reversed the decreased *Epcam* expression and the decreased percentage of EpCAM^+^ LPCs in the liver tissues of CDE diet-fed *Bex1*^−/−^ mice (Fig. 7f, g). H&E analysis and serum ALT detection demonstrated that GW9662 inhibited severe liver injury in CDE diet-fed *Bex1*^*−/−*^mice (Fig. 7h, i). These results together suggest that BEX1 inhibits PPARG to regulate LPC expansion.

## Discussion

Liver regeneration under chronic or severe liver injury is not well defined. LPCs are activated during severe and persistent liver diseases and can repopulate the liver, thus improving liver function and architecture [33]. However, the mechanisms of LPC regulation under physiological and pathological conditions are not fully understood and need to be explored. In this study, we identified BEX1 as an essential regulator of LPC activation during liver regeneration because *Bex1* deficiency severely impaired LPC expansion in CDE diet-induced liver injury by inhibiting the proliferation of and enhancing the apoptosis of LPCs. We further demonstrated that BEX1 inhibited PPARG, which contributed to the promoting effects of BEX1 on LPC expansion.

Previous studies have reported that BEX1 is highly expressed in the brain and can be detected in several peripheral tissues [34, 35]. However, in liver tissues, BEX1 is highly expressed after birth but is then subsequently downregulated, with only a marginal mRNA level detectable in adult mouse livers [35]. The *Bex1* transcript level has also been shown to be increased during hepatocyte dedifferentiation, and *Bex1* is considered a marker for hepatocyte differentiation/dedifferentiation processes and tumour formation [22]. *Bex1* expression was significantly increased in hepatocellular carcinoma cell lines compared with normal hepatocyte cell lines, promoting cell proliferation [36]. Here, we performed microarray analysis and unexpectedly identified that hepatic BEX1 expression was increased during CDE diet-induced liver injury. IF staining showed co-localized signals for EpCAM and BEX1, indicating high expression of BEX1 in LPCs. We also observed a population of BEX1^+^ hepatocytes in CDE diet-fed mice. We speculated that these cells may be newly formed hepatocytes arising from differentiated progenitors, but this requires further confirmation.

BEX1 has been demonstrated to play critical roles in muscle and neural regeneration [18–21]. Considering the high expression of BEX1 in LPCs, we hypothesized that BEX1 might be involved in regulating LPC activation during liver regeneration. As expected, *Bex1*^*−/−*^ mice demonstrated reduced LPC expansion and increased liver injury in response to a CDE diet. Proliferation and apoptosis are tightly coupled, and cell cycle regulators can influence both cell division and cell death, thereby sustaining LPC expansion. We found that BEX1 promoted LPC proliferation in vivo and in vitro. Furthermore, the contribution of BEX1 to LPC expansion occurs, at least partially, through the regulation of the cell cycle, based on the upregulation of MYC and the downregulation of CDKN1A. Simultaneously, *Bex1* deficiency enhanced etoposide-induced LPC apoptosis, while BEX1 overexpression alleviated apoptosis. Obviously, BEX1 is of great importance in the regulation of LPC expansion through regulating proliferation and apoptosis. BEX1 has been reported to be involved in nerve growth factor-mediated neural stem cell survival through cell cycle regulation [18]. Thus, it is of great interest whether BEX1 acts downstream of Wnt or FGF or other mitogens in LPCs to regulate their expansion.

Self-renewal and differentiation are two characteristics of LPCs that play critical roles in their activation and expansion during liver regeneration [3]. Our results showed that BEX1 inhibition decreased the colony formation of both LPCs and LEPCs. Regarding differentiation, it was reported that *Bex1* deficiency accelerated the neuronal differentiation of neural stem cells [18]. However, in our study, *Bex1* deficiency had no effect on the hepatic differentiation of LPCs, as characterized by the similar levels of glycogen deposition, hepatocyte markers (G-6-Pase and ALB) and cholangiocyte markers (CK-19 and CK-7). This finding indicates that there may be different regulatory mechanisms of BEX1 in different cell types.

PPARG, a member of the PPAR family belonging to the nuclear receptor superfamily, is crucial for development and functions as a lipid sensor to regulate lipid metabolism [37]. PPARG is expressed at a low level in normal human and mouse livers and plays divergent roles in the pathogenesis of liver injury [32]. Our data showed a significantly higher expression of PPARG in LPCs and liver tissues from CDE diet-fed *Bex1*^−/−^ mice than in those from CDE diet-fed WT mice. Additionally, PPARG activation with rosiglitazone in WT mice reduced LPC expansion in the CDE model, a finding that was consistent with the phenomenon reported by Cheng et al. [30] and Knight et al. [31] that PPARG can inhibit LPC growth and viability. We further explored whether PPARG was involved in BEX1-regulated LPC expansion. *Pparg* was silenced in LPCs from CDE diet-fed WT and *Bex1*^−/−^ mice, reversing the inhibited proliferation and survival of LPCs from *Bex1*^−/−^ mice, as well as increasing *Myc* expression and decreasing *Cdkn1a* mRNA expression. What is more, the PPARG antagonist GW9662 could reverse the decreased *Epcam* expression and the decreased percentage of EpCAM^+^ LPCs in the liver tissues of CDE diet-fed *Bex1*^−/−^ mice, and GW9662 could inhibit severe liver injury in CDE diet-fed *Bex1*^*−/− *^mice. Thus, mechanistically, we delineate a previously unrecognized BEX1-regulated pathway in the regulation of LPC activation and expansion through compromising PPARG signalling, promoting LPC proliferation and survival.

Knight et al. [31] demonstrated that PPARG regulated the growth and apoptosis of murine LPCs but did not affect their hepatic differentiation. This finding may explain why BEX1 inhibition has no effect on hepatic differentiation of LPCs in our experiments. PPARG has also been reported to regulate hepatocyte lipogenesis and has demonstrated a prosteatotic role in the liver because mice with hepatocyte deletion of *Pparg* were protected from high-fat diet-induced steatosis [32, 38]. In our experiment, a population of BEX1^+^ hepatocytes was observed in CDE diet-fed mice, and CDE diet-fed *Bex1*^−/−^ mice showed more serious macrovesicular steatosis. Thus, it is plausible that BEX1 regulation of PPARG may also function in hepatocytes in this injury model, a phenomenon that requires further investigation.

## Conclusions

In summary, the current study showed that hepatic BEX1 expression was increased during CDE diet-induced liver injury and was elevated to a high level, primarily in LPCs. BEX1 was required for LPC activation and liver regeneration during CDE-induced liver injury by promoting the proliferation and preventing the apoptosis of LPCs. Additionally, BEX1 was required for the colony formation of LPCs but not for their hepatic differentiation. Furthermore, these effects of BEX1 on LPCs were exerted by negatively regulating PPARG signalling. Our study provides insights into the regulation network of BEX1 for LPCs and potentially provides novel targets for liver regeneration and chronic liver disease therapies.

## Additional file

Additional file 1: **Figure S1.** Genetic ablation of *Bex1* in mice*.* Genomic DNA was isolated, and genotyping of *Bex1* performed using PCR. Primers as follows: *Bex1*–3′, TTCATTTCCCCATCTGAAAGGTCCG; *Bex1*–5′, TCCCACCTACTCACCCATCCTTCTGG; LTR-5′, AAATGGCGTTACTTAAGCTAGCTTGC. Product size for WT mice is 352 bp, and for *Bex1*^−/−^ mice is 223 bp (PDF 47 kb)

## Abbreviations

ALB: Albumin
ALT: Alanine aminotransferase
Bcl-2: B-cell lymphoma-2
BEX1: Brain-expressed X-linked 1
BrdU: Bromodeoxyuridine
CCK8: Cell Counting Kit-8
CDE: Choline-deficient, ethionine-supplemented
CK-19: Cytokeratin-19
DMSO: Dimethyl sulphoxide
EpCAM: Epithelial cell adhesion molecule
FGF: Fibroblast growth factor
G-6-Pase: Glucose-6-phosphatase
H&E: Haematoxylin and eosin
IF: Immunofluorescence
LEPC: Liver epithelial progenitor cell
LPC: Liver progenitor cell
NPC: Non-parenchymal cell
PARP: Poly ADP-ribose polymerase
PAS: Periodic acid–Schiff
PBS: Phosphate-buffered saline
PBST: PBS containing 0.1% Tween-20
PCR: Polymerase chain reaction
PFA: Paraformaldehyde
PI: Propidium iodide
PPARG: Peroxisome proliferator-activated receptor gamma
WT: Wild-type
