# Supplementary figures and images for: A pivotal role of BEX1 in liver progenitor cell expansion in mice
Source: Stem Cell Res Ther. 2018 Jun 15;9:164. doi: 10.1186/s13287-018-0905-2 (PMC6002993; doi:10.1186/s13287-018-0905-2)

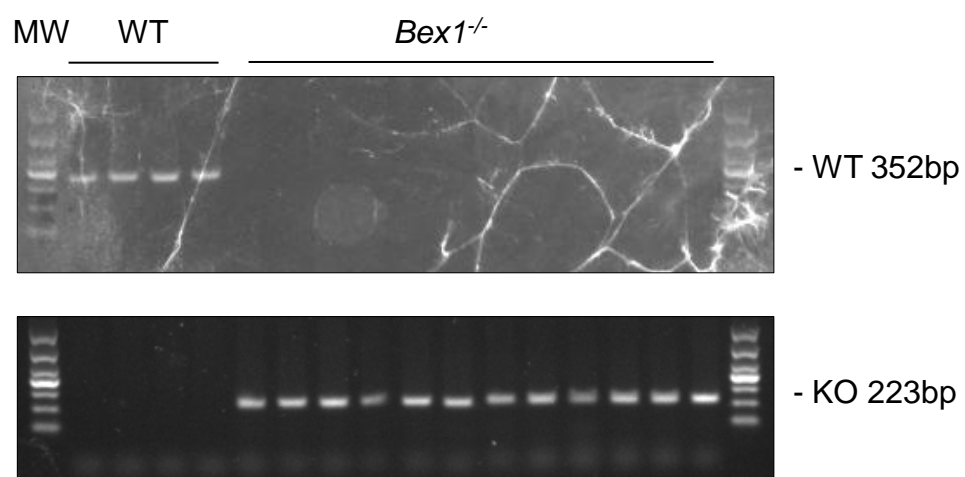

**Figure S1**

Supplement: Supplementary file 1 — Figure S1. Genetic ablation of Bex1 in mice. Genomic DNA was isolated, and genotyping of Bex1 performed using PCR. Primers as follows: Bex1–3′, TTCATTTCCCCATCTGAAAGGTCCG; Bex1–5′, TCCCACCTACTCACCCATCCTTCTGG; LTR-5′, AAATGGCGTTACTTAAGCTAGCTTGC. Product size for WT mice is 352 bp, and for Bex1−/− mice is 223 bp (PDF 47 kb) [file 13287_2018_905_MOESM1_ESM.pdf]
